# The Comparative Analysis of the Effectiveness of Four Different Storage Media (Placentrex, Propolis 10%, Pomegranate Juice 5%, and Hank's Balanced Salt Solution) in Preserving the Viability of Periodontal Ligament Cells: An In Vitro Study

**DOI:** 10.7759/cureus.42996

**Published:** 2023-08-05

**Authors:** Musaffar Thoyalil, Dhanya Kamalakshan Belchada, Konsam Bidya Devi, Rekha Vasantha Ravi, Mridhul Madathikandy Uchummal, Ramnesh Parikkal

**Affiliations:** 1 Department of Pedodontics and Preventive Dentistry, PSM Dental College, Thrissur, IND; 2 Department of Pedodontics and Preventive Dentistry, Mahe Institute of Dental Sciences and Hospital, Mahe, IND; 3 Department of Periodontology, Dental College, Jawaharlal Nehru Institute of Medical Sciences, Imphal, IND; 4 Department of Oral Pathology and Microbiology, Noorul Islam College of Dental Sciences, Trivandrum, IND

**Keywords:** placentrex, pomegranate 5%, propolis 10%, hbss, storage media, periodontal ligament cell viability

## Abstract

Background: Out of all traumas, dentoalveolar trauma occurs quite frequently and is commonly linked to avulsion injuries. Of all severe lesions to the permanent dentition, tooth avulsion accounts for 1%-16% of cases. The extraoral dry time and the storage medium in which the tooth is stored and its capacity to promote cell viability are two important variables influencing the prognosis of an avulsed tooth following replantation.

Aim: The aim of this paper is to assess and contrast the effectiveness of placentrex, propolis 10%, pomegranate juice 5%, and Hank's balanced salt solution (HBSS) as a storage medium in preserving the periodontal ligament (PDL) cells' viability.

Methods: Sixty freshly extracted premolars were put in four various media for 45 minutes each, including HBSS, placentrex, propolis 10%, and pomegranate juice 5%, and placed in an incubator for 30 minutes in 15 mL falcon tubes with 2.5 mL solutions of collagenase 0.2 mg/mL and dispase 2.4 mg/mL in phosphate-buffered saline. Bovine serum was added after incubation, and the mixture was centrifuged for four minutes. Trypan blue 0.4% was used to identify the cells. Under a light microscope, a hemocytometer was utilized to quantify the number of periodontal ligament cells that were still alive.

Results: Propolis, pomegranate, and placentrex are all significantly inferior to HBSS in the context of the sustainability of the cells. It was discovered that HBSS with placentrex and pomegranate juice 5% was statistically insignificant. The difference in periodontal ligament cell viability between propolis 10%, HBSS, placentrex, and pomegranate juice 5% was shown to be statistically significant.

Conclusion: When compared to other media such as propolis 10% and pomegranate juice 5%, placentrex is a better alternative storage medium for avulsed teeth.

## Introduction

Traumatic dental injury poses a significant challenge to overall dental health due to its frequent occurrence, impact on younger individuals, substantial costs involved, and need for lifelong care for affected patients. Avulsion injuries are among the most severe and intense types of damage to the dentition [1]. An avulsion is complicated by a substantially impaired neurovascular supply and the loss of vitality of the pulp. A vital periodontium is essential for the healthy recovery of a transplanted avulsed tooth [1]. Additionally, the oral dry time and the storage medium have an impact on the periodontal ligament (PDL) cells' ability to survive and serve as the significant elements influencing how well an avulsed tooth may heal after replantation. Replanting a tooth within five minutes will usually ensure that the PDL cells work normally again right away. The progenitor or stem cells, however, become unable to transform into fibroblasts after a dry storage period of at least 15 minutes and at least 30 minutes; it is likely that the PDL cells still present on the root surface have died [2]. As a result, the viability of the periodontium is certainly impacted by the extraoral storage conditions, and the rapid replanting of an avulsed tooth has a positive impact on PDL cell viability with a reported frequency of 85% in adults. The victim's and witnesses' emotional states, awareness of the proper course of action, and accessibility of the dental clinic in the vicinity are additional aspects that contribute to the effectiveness of the replantation procedure.

The healing reactions stimulate the growth of fibroblasts, which is essential for repair. The existence and migration of particular cell populations with regeneration capacity, such as PDL progenitor cells, along with the absence of pollutants, foreign substances, and/or microbes, are necessary for healing. After replantation, several healing patterns can occur: favorable healing with a normal periodontal ligament (PDL) and no root resorption, healing with surface-related resorption associated with the repair, and unfavorable healing characterized by ankylosis, infection, or inflammation-related resorption [3]. Due to their remarkable capacity for regeneration, dental tissues differ significantly from the majority of other tissues in the body. The release of several signals that prompt neighboring cells to proliferate, migrate, or differentiate facilitates the ability of injured tissues of the pulp/PDL to regenerate or repair with fibrous scar tissue or the bone [3].

A solution that resembles the oral environment is known as a storage medium, and it is used to retain the viability of the cells of the periodontium after avulsion [2]. To prevent ankylosis and replacement resorption, nevertheless, the capacity of a transport medium is more crucial. Numerous investigations have examined the effectiveness of different storage media, such as saline, tap water, milk, saliva, Hank's balanced salt solution (HBSS), Viaspan, Gatorade, propolis, culture media, growth factors, ascorbic acid, levodopa (L-DOPA), cryoprotective agents, catalase supplementation, and green tea extract, in restoring the viability of the periodontium of an avulsed tooth [1].

When a tooth, which has been subjected to an extended period of extraoral exposure, is replanted into its socket using an optimal storage medium, it exhibits favorable healing outcomes. Tooth replantation refers to the act of reinserting a tooth into its socket after it has been forcefully dislodged or avulsed [4]. After being stored in appropriate storage settings for 1-3 hours, an avulsed tooth could be successfully replanted with no further concerns (Özan et al.). Cemental PDL fiber degeneration is a frequent occurrence, and the necrotic remnants of the periodontium on the root augment the inflammatory root resorption, leading to the loss of replanted teeth, which usually occurs between one and four hours following avulsion [4]. The aim of the study was to compare the effectiveness of four different storage media, placentrex, propolis 10%, pomegranate juice 5%, and Hank's balanced salt solution, in preserving the viability of the PDL cells.

## Materials and methods

Sixty newly extracted human premolar teeth from the Oral and Maxillofacial Surgery Department at Kurunji Venkatramana Gowda (KVG) Dental College in Sullia, which had normal periodontium and closed apices, were chosen. For the study, 60 healthy premolar teeth that were extracted for orthodontic treatment were chosen. For 30 minutes, all the teeth will be allowed to dry in order to mimic a clinical setting. Following collection, the teeth were sorted into four groups of 15 at random. Ethical clearance was obtained from KVG Medical College and Hospital with institutional review board number IEC/19/11/2015.

The inclusion criteria include sixty freshly extracted upper or lower first or second premolar teeth for orthodontic purposes, upper or lower first and second premolar teeth with closed apices, non-carious premolar, and teeth without periodontal diseases. The exclusion criteria include teeth that have moderate to severe gum disease, teeth with broken roots or crowns, and carious teeth.

The materials used were Hank's balanced salt solution (HBSS), placentrex, pomegranate juice 5%, propolis 10%, collagenase, dispase, bovine serum, phosphate-buffered saline, and trypan blue. An armamentarium such as curette, falcon tubes, Neubauer chamber, micropipette, light microscope with a hemocytometer, and centrifugation machine was used.

Sixty recently extracted human premolar teeth were obtained, all of which had closed apices and a normal periodontium. The injured cells were removed from the coronal 3 mm of the periodontal ligament using a curette. Fifteen teeth were distributed randomly among the four groups. The teeth in the test groups were allowed to dry for half an hour before being submerged for 45 minutes in one of the test storage media listed as follows: group I, specimens kept in HBSS; group II, specimens kept in placentrex; group III, specimens kept in propolis 10%; and group IV, specimens kept in pomegranate juice 5%. The following products are used: HBSS, placentrex, and propolis 10%. Pomegranate juice preparation of 5%, red pulp, and/or red skins from fresh pomegranate fruits was gathered. The quantity needed for this investigation was prepared, and the grains were thoroughly sorted to draw the juice out of them. Squeezing the grains and filtering the juice produced the pomegranate juice. The rotary flash evaporator was used to condense pure juice to the ideal level. Pomegranate juice 5% concentration allows for the effective evaluation of the bioactive compounds present in pomegranate without excessive dilution or concentration, ensuring reliable results. Its seeds and juice contain all the medicinal properties of the fruit. It supports fibroblast cell attachment and proliferation and has anti-inflammatory, anti-carcinogenic, and antioxidant properties [5].

**Figure 1:**
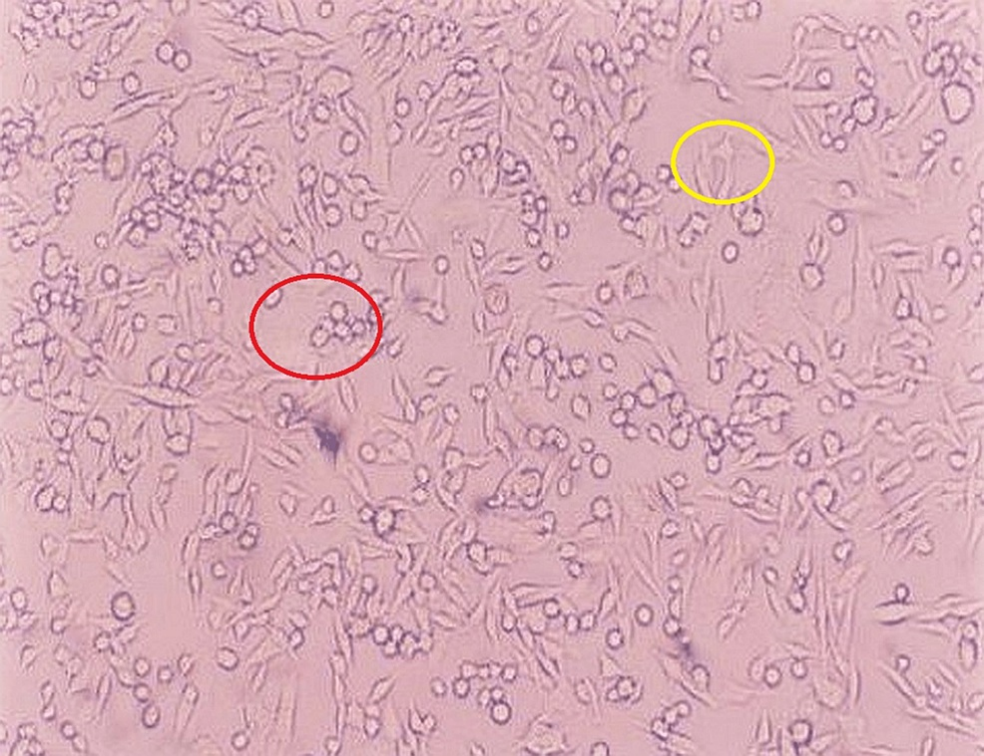
HBSS medium viable and non-viable cells Red circle: viable cells. Yellow circle: non-viable cells HBSS: Hank's balanced salt solution

**Figure 2:**
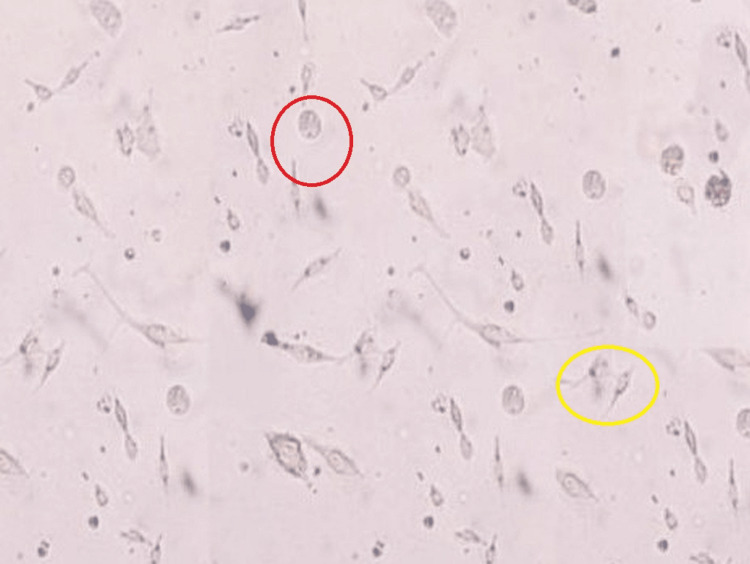
Placentrex medium with cell culture Red circle: viable cells. Yellow circle: non-viable cells

**Figure 3:**
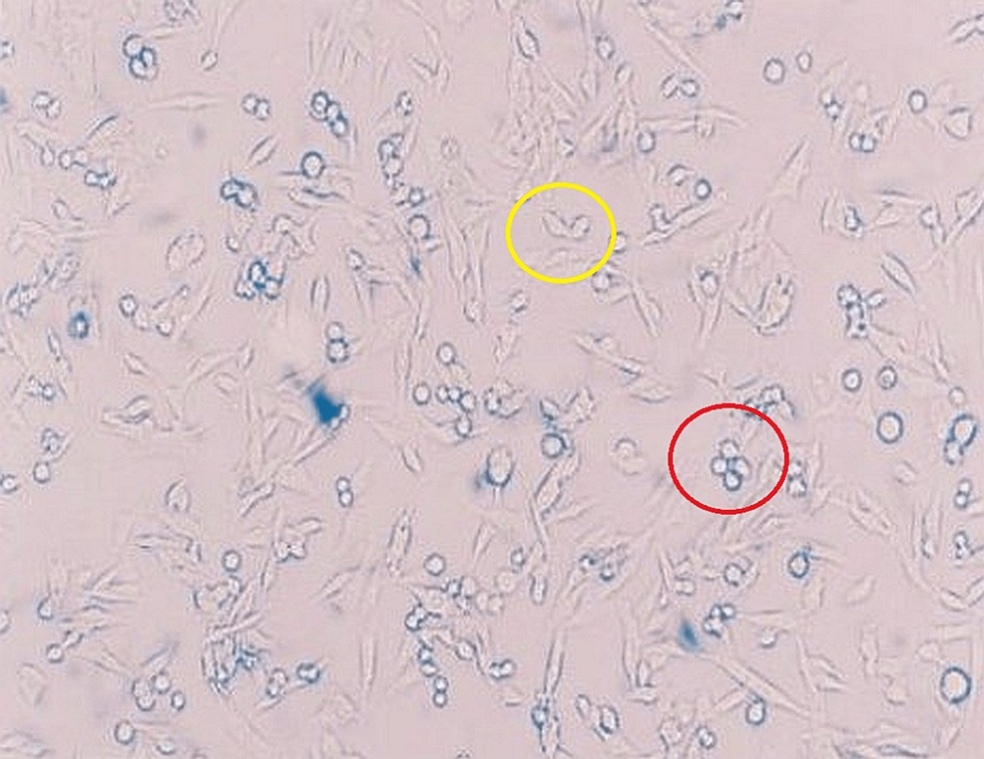
Propolis medium with cell culture Red circle: viable cells. Yellow circle: non-viable cells

**Figure 4:**
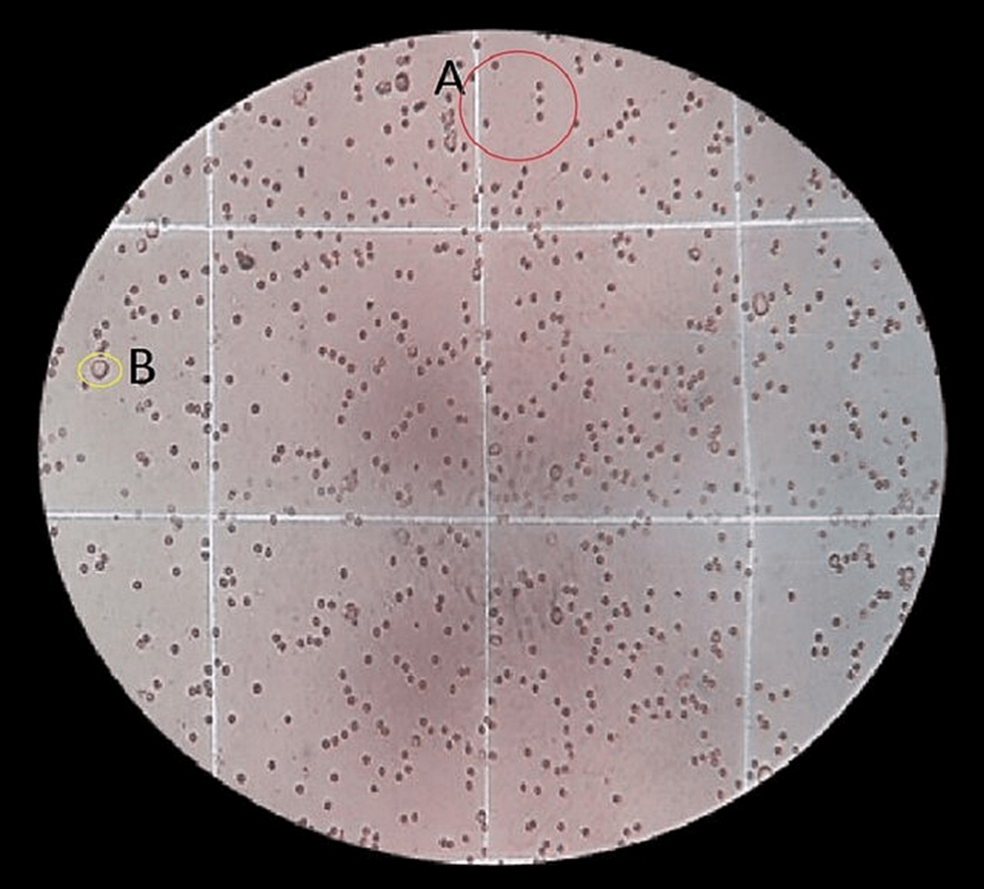
Pomegranate medium with cell culture (A) Red circle: viable cells. (B) Yellow circle: non-viable cells

Each experimental tooth was dried and placed in the storage media and placed in the incubator for 30 minutes in 15 mL falcon tubes with 2.5 mL solutions of collagenase and dispase in phosphate-buffered saline. Then, 50 L of bovine serum was pipetted into all the tubes. The supernatant was extracted using sterile micropipettes after being centrifuged for four minutes. Trypan blue-labeled cells were employed to determine cell vitality. The intact cell membranes found in living cells prevent some dyes from penetrating. This test involves adding dye to a cell solution, which is then examined visually to determine if the cells have absorbed or rejected the dye. The amount of periodontal ligament cells that are still alive is determined by a hemocytometer under a 40× light microscope. We examined the ability of cells to absorb stains. Viable cells have transparent cytoplasm as opposed to non-viable cells, which have blue cytoplasm. Cell counting was done using the following formulae: total cells - stained cells × 100, and viability percentage = viable cells × 100 / total cells (Figures 1-4).

One-way analysis of variance (ANOVA) and Tukey's post hoc tests were applied to analyze the acquired data after it had been imported into a Microsoft Excel spreadsheet (Microsoft® Corp., Redmond, WA).

## Results

**Figure 5:**
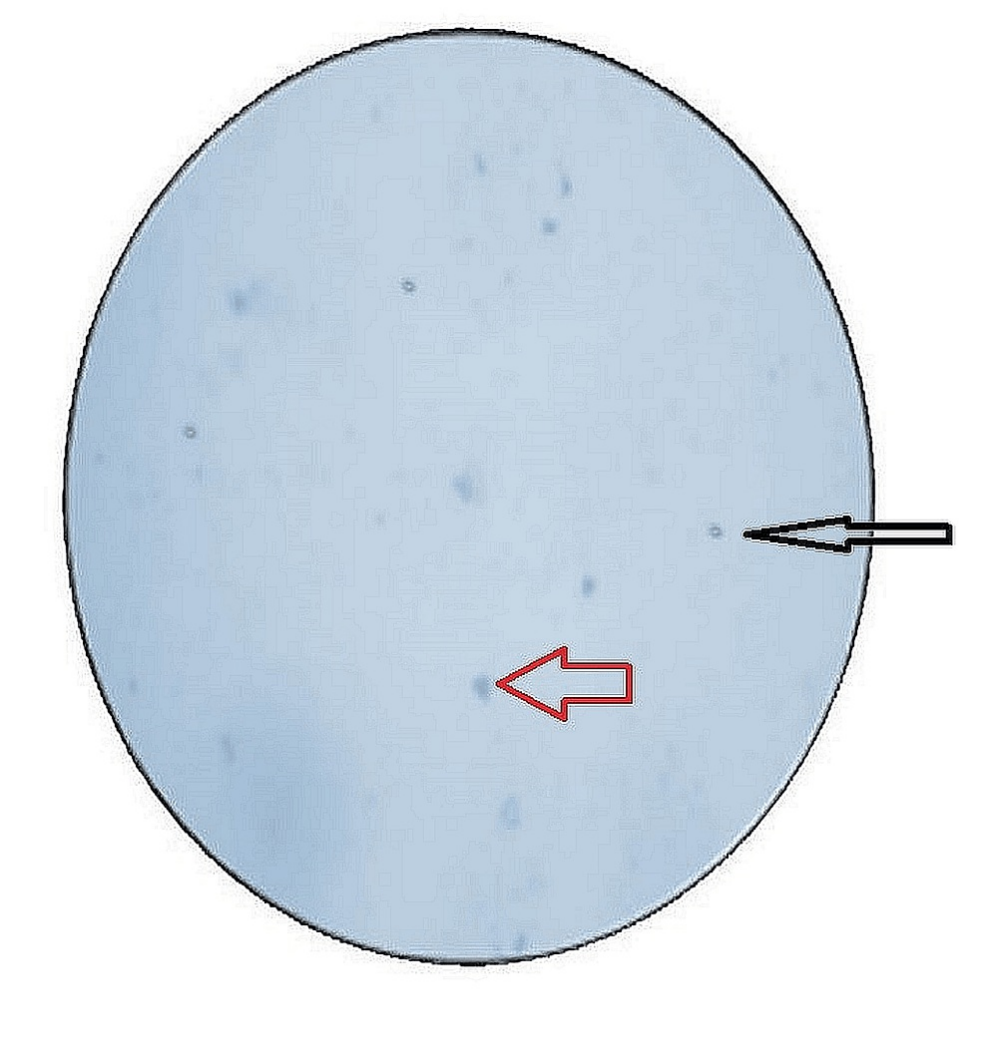
Microscopic image of the viable cells Black arrow: non-viable cells of the periodontal ligament. Red arrow: viable cells of the periodontal ligament

Descriptive information for the viability of the periodontal ligament (PDL) cells in various storage media was displayed as percentages, a mean, and a standard deviation (Table 1 and Figure 5).

**Table 1:** Descriptive statistics and one-way ANOVA for the viability of the periodontal ligament (PDL) cells in various storage media HBSS, Hank's balanced salt solution; SD, standard deviation; N, number of teeth; ANOVA, analysis of variance

Medium	N	Mean	SD	p-value
Propolis 10%	15	50.81	16.96	<0.001
HBSS	15	73.12	7.41	
Placentrex	15	69.14	7.82	
Pomegranate juice 5%	15	62.94	9.64	
Total	60	64.00	13.79	

Tukey's post hoc multiple comparison tests revealed a statistically significant difference in periodontal ligament cell viability between propolis 10% and HBSS (p < 0.001), propolis 10% and placentrex (p = 0.001), propolis 10% and pomegranate juice 5% (p = 0.021) (Table 2).

**Table 2:** Tukey's post hoc test for periodontal ligament cell viability HBSS: Hank's balanced salt solution

Storage medium	Other storage media	Mean difference	Standard error	p-value
HBSS	Placentrex	3.985	4.07	0.762
	Propolis 10%	22.32	4.07	0.001
	Pomegranate juice 5%	10.18	4.07	0.070
Placentrex	HBSS	-3.98	4.07	0.762
	Propolis 10%	18.33	4.07	0.001
	Pomegranate juice 5%	6.20	4.07	0.431
Propolis 10%	HBSS	-22.32	4.07	0.001
	Placentrex	-18.33	4.07	0.001
	Pomegranate juice 5%	-12.13	4.07	0.021
Pomegranate juice 5%	HBSS	-10.18	4.07	0.070
	Placentrex	-6.20	4.07	0.431
	Propolis 10%	12.13	4.07	0.021

The descriptive statistics were used to check for the mean number of viable periodontal cells in various storage media. It was found that the HBSS group contained more number of live cells (3.80 ± 1.21) compared to placentrex, propolis 10%, and pomegranate juice 5%. One-way ANOVA revealed a statistically highly significant difference between HBSS, placentrex, propolis 10%, and pomegranate juice 5% (p = 0.001) (Table 3).

**Table 3:** Descriptive statistics and ANOVA table for mean viable periodontal ligament cells HBSS, Hank's balanced salt solution; SD: standard deviation; N, number of teeth; ANOVA, analysis of variance

Medium	N	Mean	SD	p-value
HBSS	15	3.80	1.21	0.001
Placentrex	15	3.67	1.11	
Propolis 10%	15	2.00	1.25	
Pomegranate juice 5%	15	3.00	1.60	

Tukey's post hoc multiple comparison test revealed a statistically highly significant difference between HBSS and propolis 10% (p = 0.002). On the contrary, the difference between HBSS and placentrex (p = 0.992) and HBSS and pomegranate juice 5% was discovered to be statistically insignificant (p = 0.346). In terms of periodontal ligament cell viability, the difference between propolis 10% with placentrex was shown to be statistically significant (p = 0.005) (Table 4).

**Table 4:** Tukey's post hoc test for live periodontal ligament cells HBSS: Hank's balanced salt solution

Storage medium	Other storage media	Mean difference	Standard error	Significance
HBSS	Placentrex	0.13	0.48	0.992
	Propolis 10%	1.80	0.47	0.002
	Pomegranate juice 5%	0.80	0.47	0.346
Placentrex	HBSS	-0.13	0.48	0.992
	Propolis 10%	1.67	0.47	0.005
	Pomegranate juice 5%	0.67	0.47	0.507
Propolis 10%	HBSS	-1.80	0.47	0.002
	Placentrex	-1.67	0.47	0.005
	Pomegranate juice 5%	-1.00	0.47	0.167
Pomegranate juice 5%	HBSS	-0.80	0.47	0.346
	Placentrex	-0.67	0.47	0.507
	Propolis 10%	1.00	0.47	0.167

The descriptive statistics for assessing non-viable periodontal ligament cells found that pomegranate juice 5% had a greater number of mean non-viable cells (2.33 ± 1.05). HBSS showed the least number of periodontal ligament dead cells (1.80 ± 1.08) compared to other storage media. However, one-way ANOVA disclosed a statistically insignificant difference between the different groups (Table 5).

**Table 5:** Descriptive statistics and ANOVA table for assessing non-viable periodontal ligament cells HBSS: Hank's balanced salt solution; SD, standard deviation; N, number of teeth; ANOVA, analysis of variance

Medium	N	Mean	SD	p-value
HBSS	15	1.80	1.08	0.54
Placentrex	15	2.07	1.03	
Propolis 10%	15	2.27	1.22	
Pomegranate juice 5%	15	2.33	1.05	
Total	60	2.12	1.09	

Tukey's post hoc multiple comparison tests were utilized for assessing non-viable periodontal ligament cells. While comparing HBSS, placentrex, propolis 10%, and pomegranate juice 5% in terms of number of dead cells, the difference was found to be statistically insignificant (p > 0.05) (Table 6).

**Table 6:** Tukey's post hoc test for assessing non-viable periodontal ligament cells HBSS: Hank's balanced salt solution

Storage medium	Other storage media	Mean difference	Standard error	p-value
HBSS	Placentrex	-0.27	0.401	0.910
	Propolis 10%	-0.47	0.401	0.652
	Pomegranate juice 5%	-0.53	0.401	0.548
Placentrex	HBSS	0.27	0.401	0.910
	Propolis 10%	-0.20	0.401	0.959
	Pomegranate juice 5%	-0.27	0.401	0.910
Propolis 10%	HBSS	0.47	0.401	0.652
	Placentrex	0.20	0.401	0.959
	Pomegranate juice 5%	-0.07	0.401	0.998
Pomegranate juice 5%	HBSS	0.53	0.401	0.548
	Placentrex	0.27	0.401	0.910
	Propolis 10%	0.07	0.401	0.998

## Discussion

Clinical studies suggest that tooth fractures occurring due to trauma in kids and teens are a widespread issue, and numerous researches have revealed an increase in the occurrence of these injuries [5]. The most common tooth to suffer an avulsion injury is the maxillary central incisor, which is the worst type of dentoalveolar injury. The ability of each cell in the affected tissues, including the pulp and PDL cells, to recover will determine the regrowth trend of an avulsed tooth after transplantation. The prognosis of a transplanted tooth relies on the extent of damage sustained by the periodontal ligament, which plays a vital role in the regeneration of the periodontium and in preventing tooth resorption [1].

The key factors that significantly affect the healing of the PDL following replantation include the severity of the trauma caused to the root, the length of extraoral storage, and the kind of medium utilized to store the avulsed tooth. Cells from the normal periodontal ligament have a slightly alkaline pH and an osmolality of 320 mOsm/kg [6]. Cells may live for a long time between pH 6.6 and 7.8, but their growth is best between pH 7.2 and 7.4 [7]. The storage medium of the tooth functions as an excellent transport system for the tooth as well as a means of preserving PDL cell health. The nutrients required for the periodontal ligament cells to persevere are provided by the gingival crevicular fluid to the tooth. The delivery of essential metabolites is necessary for the PDL to remain attached to the traumatized root. As a result, the storage medium needs to be full of nutrients that the PDL cells can utilize to grow.

When the supply of vital metabolites is cut off, cell death occurs. The primary tenet of this viability may entail inhibiting bacterial protein production, promoting the activity of fibroblasts, and promoting connective tissue regeneration, all of which help the periodontal ligament mend after injury [8]. Studies have demonstrated that after 1-3 hours of being kept in the appropriate preserving circumstances, an avulsed tooth can be successfully replanted [9]. A storage medium that can retain the viability of the periodontium, mitogenicity, and clonogenicity would be ideal since it would make it easier for cells to regenerate on the root surface, which would stop future root resorption. The perfect storage medium should also be simple to access in case of an emergency. The first researchers to examine the preservation outcome of milk and saliva on periodontium were Blomlöf and Otteskog [10]. Beginning in the 1980s, additional research that was related to the original study indicated that milk was more effective than saliva in terms of the quantity of viability of the cells, its size, and its capacity to repair experimentally induced trauma.

As per research, for short-term storage, saliva works well. However, as saliva might harm PDL cells, it should not be utilized to store the tooth for longer than an hour [11]. Due to the presence of bacteria and relatively low osmolality (60-70 mOsm/kg), which causes inflammation and injury to the membrane in the PDL cells, an avulsed tooth cannot be kept in saliva for an extended period of time [11]. In a study, it was shown that oral rehydration solution and HBSS both maintained PDL cell viability and milk, and both did it better [12]. Fresh human placental extract and nitrogen are combined to make placentrex. As a result of its anti-inflammatory and antiaging qualities, it can be utilized to treat a variety of ailments. It can aid in enhancing blood flow, elevating hormone levels, and accelerating tissue regeneration to promote wound healing with the least amount of scarring. De et al. used zymography to demonstrate that the extract has significant gelatinase/collagenase activity and showed that a collagenase-active protein was derived from human placental extract [13]. The thermostability of the intrinsic collagenase activity of placental extract may have broad physiological implications in remodelling and destroying collagen in the context of wound healing and inflammation.

Propolis is typically composed of 50% resinous vegetable balsam, 30% wax, 10% aromatic and essential oils, 5% pollen, and several additional materials, such as organic debris, at a ratio of 5%. It was reportedly more effective than HBSS or milk (Martin et al.) [1]. A statistically significant outcome was revealed in the current study when comparing propolis 10% with HBSS, placentrex, and pomegranate juice 5% in terms of the viable PDL cells. In the present study, the viability of the periodontal ligament (PDL) cells was compared among different storage media, including propolis 10%, HBSS, placentrex, and pomegranate juice 5%. Placentrex, HBSS, and pomegranate juice 5% did not show statistically significant differences (p > 0.005) in sustaining PDL viability. However, propolis exhibited a significantly higher preservation of PDL viability (p < 0.005) compared to the other storage media in the study. Of the four media, HBSS revealed the greatest number of periodontal ligament cells that were still alive. Compared to propolis 10% and pomegranate juice 5%, placentrex preserved the viability of the periodontium effectively.

It was documented that propolis 10% and Dulbecco's Modified Eagle's Medium (DMEM) worked better together than propolis 20% and DMEM. This indicates that the concentration of propolis 20% has some cytostatic/cytotoxic effects on the cells [14]. The viability of the periodontium and dental pulp cells at a lower concentration of propolis (10%) was found to maintain the cell viability greater than 75% with a lower toxicity in a prior study [15]. Furthermore, it should be noted that high concentrations of propolis have been found to exhibit cytotoxic effects on gingival fibroblasts [16]. Propolis was utilized at 10% and 20% by Özan et al. [9], and they discovered that propolis 10% worked better as storage than propolis 20%. Martin and Pileggi [17] showed that propolis (50% and 100%) restored the viability of the periodontium considerably than HBSS, which is contrary to the present study. This can be attributed to the varying concentrations and solid form of propolis utilized in their study.

Pomegranate is a fruit with significant antioxidant qualities. The polyphenols in pomegranate juice, notably punicalagin, the main fruit ellagitannin, and ellagic acid (EA), are thought to be responsible for the fruit's strong antioxidant activity. The main antioxidant polyphenol present in pomegranate juice is punicalagin, serving a crucial role in retaining the viability of the periodontium. Pomegranate polyphenols and flavonoids both have antioxidant and antiviral effects, which may increase the viability of PDL cells. Flavonoids from pomegranates have microbicidal and anti-inflammatory properties [18].

The advantages of HBSS is that it gives an ionic balance. HBSS is a physiologically balanced solution of salts and minerals, which helps maintain the osmotic balance and pH of the culture environment, ensuring cell viability and functionality. It is devoid of nutrients, growth factors, and serum, which minimizes interference with the experimental conditions, making it suitable for certain specific research applications. HBSS is ideal for short-term cell preservation, as it keeps the cells alive without promoting proliferation. The limitations of HBSS include that it lacks growth factors and other components necessary for sustained cell growth and differentiation, making it unsuitable for long-term culture experiments.

The advantage of placentrex is that it contains various growth factors, cytokines, and proteins that support cell proliferation, migration, and differentiation. This rich composition promotes the overall health and growth of the PDL cells. Placentrex helps maintain high cell viability, enhancing the success of long-term culture studies. The limitation of placentrex is that the use of human placental extract may raise ethical considerations and limit its availability in certain regions or research institutions.

The advantage of propolis media is that it contains antioxidants that protect cells from oxidative stress, supporting cell health and longevity. Limitations include that its composition can vary depending on its source and harvesting conditions, leading to inconsistencies in experimental results, and propolis may not provide a comprehensive set of growth factors and nutrients required for robust long-term cell culture.

The advantages of pomegranate media is that it contains antioxidants, such as polyphenols, which can protect cells from oxidative damage and maintain their health. Pomegranate has anti-inflammatory properties that may benefit the PDL cells by reducing inflammation in the culture environment and has potential pro-proliferative effects. The limitations of pomegranate media is that PDL cell culture may not have been extensively studied, leading to a lack of comprehensive data on its effectiveness, and may be more expensive and less readily available than standard culture media.

In the current investigation, the preservation of periodontal ligament cells' viability in pomegranate juice was a statistically insignificant finding (p > 0.05) when compared to HBSS and placentrex but statistically significant (p = 0.021) when compared to propolis. This is in line with a study by Tavassoli-Hojjati et al. that discovered that pomegranate influences the proliferation of fibroblast cells [19]. At lower concentrations of 1% and 2.5%, this proliferative effect is only present for an hour, and a more favorable effect is noticed at a higher concentration. At six hours, cell viability has increased to its highest level. It also encourages firmly attached cells. Therefore, it could be a useful storing medium. Further research is required because there has not been much done to evaluate its effectiveness. Hank's balanced salt solution (HBSS) medium was chosen for this investigation since it is advised as the recommended storage solution by the American Association of Endodontists (AAE). In our investigation, HBSS showed a more number of live periodontal ligament cells than did propolis 10%, placentrex, or pomegranate juice 5%, and the differentiating factor among HBSS and propolis 10% was statistically significant (p = 0.002). This is consistent with a research by Nozari et al., which found that HBSS maintained PDL cell viability better than honey milk and milk [20].

The findings of the research demonstrated that the HBSS group poses remarkably greater amount of viable periodontal ligament cells than other groups; placentrex demonstrated much more viable periodontal ligament cells than pomegranate juice 5% and propolis 10%. However, more extensive investigations and research are needed to confirm the advantages of HBSS and placentrex as suitable storage media.

There are several limitations; the study used only 60 freshly extracted premolars, which may not provide a comprehensive representation of the general population. A larger sample size would have increased the statistical power and generalizability of the findings. The experiment was conducted using extracted premolars and not in a clinical setting involving actual avulsed teeth. The results may not accurately reflect the conditions and outcomes of tooth avulsion in real-life situations. The long-term viability and potential for successful replantation were not considered. Tooth avulsion requires the long-term preservation of cell viability for successful outcomes, and the study did not investigate this aspect. Evaluating the success of the storage media in terms of clinical outcomes would provide a more comprehensive understanding of their effectiveness. Using additional evaluation methods, such as gene expression analysis or histological examination, would have provided a more comprehensive assessment of cell viability. The specific characteristics of the sample population, such as age, gender, or underlying oral health conditions, could significantly influence the results and limit the generalizability of the findings to other populations.

## Conclusions

According to the study's findings, the HBSS group had a considerably larger percentage of live periodontal ligament cells than the placentrex group. Placentrex should be contrasted favorably with other media, such as propolis 10% and pomegranate juice 5%, as a viable mode of transport for avulsed teeth. However, more investigation is necessary to confirm placentrex's advantages as a suitable storage medium.
